# Processing of Emotions in Speech in Forensic Patients With Schizophrenia: Impairments in Identification, Selective Attention, and Integration of Speech Channels

**DOI:** 10.3389/fpsyt.2020.601763

**Published:** 2020-11-13

**Authors:** Rotem Leshem, Michal Icht, Roni Bentzur, Boaz M. Ben-David

**Affiliations:** ^1^Department of Criminology, Bar-Ilan University, Ramat Gan, Israel; ^2^Department of Communication Disorders, Ariel University, Ariel, Israel; ^3^Psychiatric Division, Sheba Medical Center, Tel Hashomer, Israel; ^4^Baruch Ivcher School of Psychology, Interdisciplinary Center (IDC), Herzliya, Israel; ^5^Department of Speech-Language Pathology, University of Toronto, Toronto, ON, Canada; ^6^Toronto Rehabilitation Institute, University Health Networks (UHN), Toronto, ON, Canada

**Keywords:** forensic patients with schizophrenia, emotions, speech processing, selective attention, prosody, cognition

## Abstract

Individuals with schizophrenia show deficits in recognition of emotions which may increase the risk of violence. This study explored how forensic patients with schizophrenia process spoken emotion by: (a) identifying emotions expressed in prosodic and semantic content separately, (b) selectively attending to one speech channel while ignoring the other, and (c) integrating the prosodic and the semantic channels, compared to non-clinical controls. Twenty-one forensic patients with schizophrenia and 21 matched controls listened to sentences conveying four emotions (anger, happiness, sadness, and neutrality) presented in semantic or prosodic channels, in different combinations. They were asked to rate how much they agreed that the sentences conveyed a predefined emotion, focusing on one channel or on the sentence as a whole. Forensic patients with schizophrenia performed with intact identification and integration of spoken emotions, but their ratings indicated reduced discrimination, larger failures of selective attention, and under-ratings of negative emotions, compared to controls. This finding doesn't support previous reports of an inclination to interpret social situations in a negative way among individuals with schizophrenia. Finally, current results may guide rehabilitation approaches matched to the pattern of auditory emotional processing presented by forensic patients with schizophrenia, improving social interactions and quality of life.

## Introduction

Schizophrenia is a severe mental disorder that involves a wide range of deficits in cognitive, perceptual, and emotional processes (1–5). Individuals with schizophrenia show deficiencies in different dimensions of social cognition, characterized by an impaired ability to decode (perceive) verbal and non-verbal emotional expressions. In many studies, they have been reported to misattribute negative valence to ambiguous or neutral stimuli (6–12). These tendencies could heighten the risk of violence in schizophrenia (13). Indeed, individuals with schizophrenia are four to six times more likely to commit a violent crime than individuals without schizophrenia (14, 15). This group of violent offenders who have been diagnosed with schizophrenia (hereafter referred to as “forensic patients”) are the focus of interest in both research and prevention efforts in recent years (16, 17).

The current study explores whether forensic patients with schizophrenia process spoken emotion in a similar fashion as their non-clinical peers. Specifically, we target the ability to identify and integrate the emotional content of semantics (literal content) and prosody (tone of speech) of spoken sentences. There is previous evidence in the literature to suggest reduced emotional processing of semantics and prosody both in individuals with schizophrenia and in violent offenders. To the best of our knowledge, no study to date has specifically tested processing emotional content and prosody in spoken sentences among the intersecting population of violent offenders with schizophrenia. Furthermore, the majority of research tools used thus far with this clinical population did not directly assess the integration of information in both auditory channels, a routine task in daily social interactions. The current study attempts to address that gap in the existing research.

### Perception of Social Cues in Schizophrenia and Violent Behavior

The relationship between psychotic disorders and violent behaviors is complex and inconclusive (18, 19). Psychotic disorders (including schizophrenia) form the most notable group of disorders in forensic psychiatry services (20), with over 70% of men in high-security hospitals falling within this diagnostic group (21). Of psychotic disorders, schizophrenia is notable, with a high estimated prevalence rate of violent behaviors, ranging from 15.3 to 19.1% in this population [13; (22, 23)].

Research has identified multiple risk factors for aggressive and violent behavior related to schizophrenia (23–25). Deficits in affective processing are suggested as one of the main precursors to violent behavior (26). This type of difficulty is also one of the key features of schizophrenia as defined by the DSM [DSM-5, (27)] and has been identified in various studies [e.g., (6, 28, 29)]. Specifically, individuals with schizophrenia demonstrate problems in the perception of emotional material, verbal as well as non-verbal (6, 7), and they tend to misidentify neutral cues as negatively-valenced (30). For example, patients with schizophrenia have been found to be poorer than controls at recognizing emotions in facial expressions, and have misattributed emotions to neutral expressions (10). As suggested by Weiss et al. (31), misinterpretation of social emotional cues (e.g., angry or fearful facial expressions) along with a negative bias (the tendency to negatively interpret social situations) impairs adaptive behavior in daily life situations. This, in turn, may increase the risk of violent and criminal behavior in schizophrenia (13).

### Perception of Emotions in Speech

Spoken communication, and specifically the processing of emotions in spoken language, have an important role in daily social interactions (32, 33). Spoken emotion processing is crucial for the apprehension of other's feelings and development of empathy, which in turn can dampen violence toward another person (8, 12, 34–36). Indeed, when the listener does not fully apprehend the emotion conveyed by the speaker, miscommunication can ensue, with possible negative implications for the quality of social interactions (37) and aggressive and violent behavior.

The perception of spoken emotions involves the integration of several modalities, including visual and auditory channels. In the absence of visual cues (e.g., when talking over the phone) or when visual information is degraded [e.g., due to visual sensory degradation: (38, 39); or due to visual processing impairments that are well-established in schizophrenia: (40)], the ability to derive emotional meaning in spoken language relies on how it is conveyed in two auditory speech channels—the semantic channel (the meaning of the words) and the prosodic channel (the tone of speech, intonation of voice, and indexical cues).

The literature has identified three main components of processing emotional speech in healthy young adults (32, 41–43): (a) *Identification of emotions*. Listeners successfully identify the emotions expressed in the semantic and prosodic content when presented separately; (b) *Selective attention*. Listeners fail to selectively attend to one auditory channel while actively ignoring the other, when the task calls for it; (c) *Channel integration*. Listeners process the emotional content as a whole, affected by the emotions conveyed in both the prosodic and semantic channels. Most notably, the prosody of speech appears to have a much larger impact on emotional judgment than semantics [see, (44, 45)]. Let us now briefly describe what is currently known of these components among forensic patients with schizophrenia.

### Identification of Emotions in Forensic Patients With Schizophrenia

Restricted identification of emotions has been well-documented in schizophrenia (46). These deficits were documented not only in the visual modality [facial emotion recognition; e.g., (8)], but also in the auditory modality. Specifically, there is evidence to suggest deficits in identification of emotional prosodies (9) in both pre-attentive and attentive processes [for a review, see (47)]. These were more predominately reported among male patients (48), with specific difficulties in processing negative emotions [sadness, fear, anger; (49)]. Deficits in prosodic processing for patients with schizophrenia were attributed by some researchers to early auditory dysfunction, such as deficits in basic pitch perception and auditory sensory memory (50–52).

Only a limited number of studies investigated the identification of emotional semantic content in schizophrenia (53), generally reporting impairments (1, 54). For example, when asked to identify the semantic emotions of spoken sentences pronounced with neutral prosody, patients with schizophrenia made more errors than controls [(55); averaging across study conditions].

Deficits in identification of spoken emotions (semantics and prosody) for people with schizophrenia were associated with impaired social functioning (56, 57). However, to the best of our knowledge, the literature is silent regarding identification of spoken emotions by forensic patients with schizophrenia. Most studies that tested this population focused on recognition of emotional facial expressions, indicating consistent impairments (13, 18, 31, 58–60). The current study aims to test the identification of prosodic and semantic emotions separately in this population.

### Selective Attention in Forensic Patients With Schizophrenia

Attentional deficits, specifically in selective attention, are typical of schizophrenia. These have been identified mainly via research utilizing the visual color-word Stroop test [e.g., (61–63)]. Inflated Stroop effects in schizophrenia reflect a failure to inhibit the salient, yet irrelevant, channel (word semantics) while focusing on the less salient, yet relevant, channel [word font color; for a discussion on the nature of Stroop effects in clinical populations, see (39, 64, 65)]. Another line of research tested selective attention using cross-modality visual-auditory stimuli. Larger failures of selective attention were documented for individuals with schizophrenia, with a complex effect of emotional voice on facial expression processing [(66, 67); for a review, see (47)]. These failures appear to occur already at the perceptual level, with information leakage from one channel to the other [see (68)]. There are only a few studies that tested inhibition deficits in the auditory domain alone (unimodality) for individuals with schizophrenia. Presented with spoken emotion sentences (1, 55), individuals with schizophrenia showed larger failures than controls to selectively attend to one channel (semantics or prosody) while ignoring the other.

Deficits in selective attention and inhibition of irrelevant information may (at least partly) explain violent behaviors in individuals with schizophrenia. Within the context of criminal behavior, selective attention has been associated with behavioral regulation (58, 69, 70). Accordingly, responding to social situations in a flexible and adaptive manner involves efficient inhibition of irrelevant information. Failing to ignore an irrelevant emotional cue, specifically in social situations, may lead to an inappropriate or extreme reaction, including aggressive behavior [(71); with incarcerated offenders, (58)]. For example, recidivism of aggressive behavior was found to be related to reduced selective attention among forensic patients with schizophrenia (72). The current study tests whether this subgroup performs differently in selective attention and inhibition of emotional speech channels (prosodic and semantic emotions) than controls.

### Integration of Channels in Forensic Patients With Schizophrenia

Many daily situations involve the integration of information conveyed concurrently by multiple sensory channels, e.g., visual and auditory. For example, processing emotional face-voice information involves the integration of affective cues conveyed by the two sensory modalities into a unified, multisensory percept (73). Impairment of multisensory integration is a well-known characteristic of schizophrenia (74–76). However, to the best of our knowledge, integration across auditory channels, in general, or of prosodic and semantic content, specifically, has not yet been examined in forensic patients with schizophrenia.

### The Current Study

**Figure 1:**
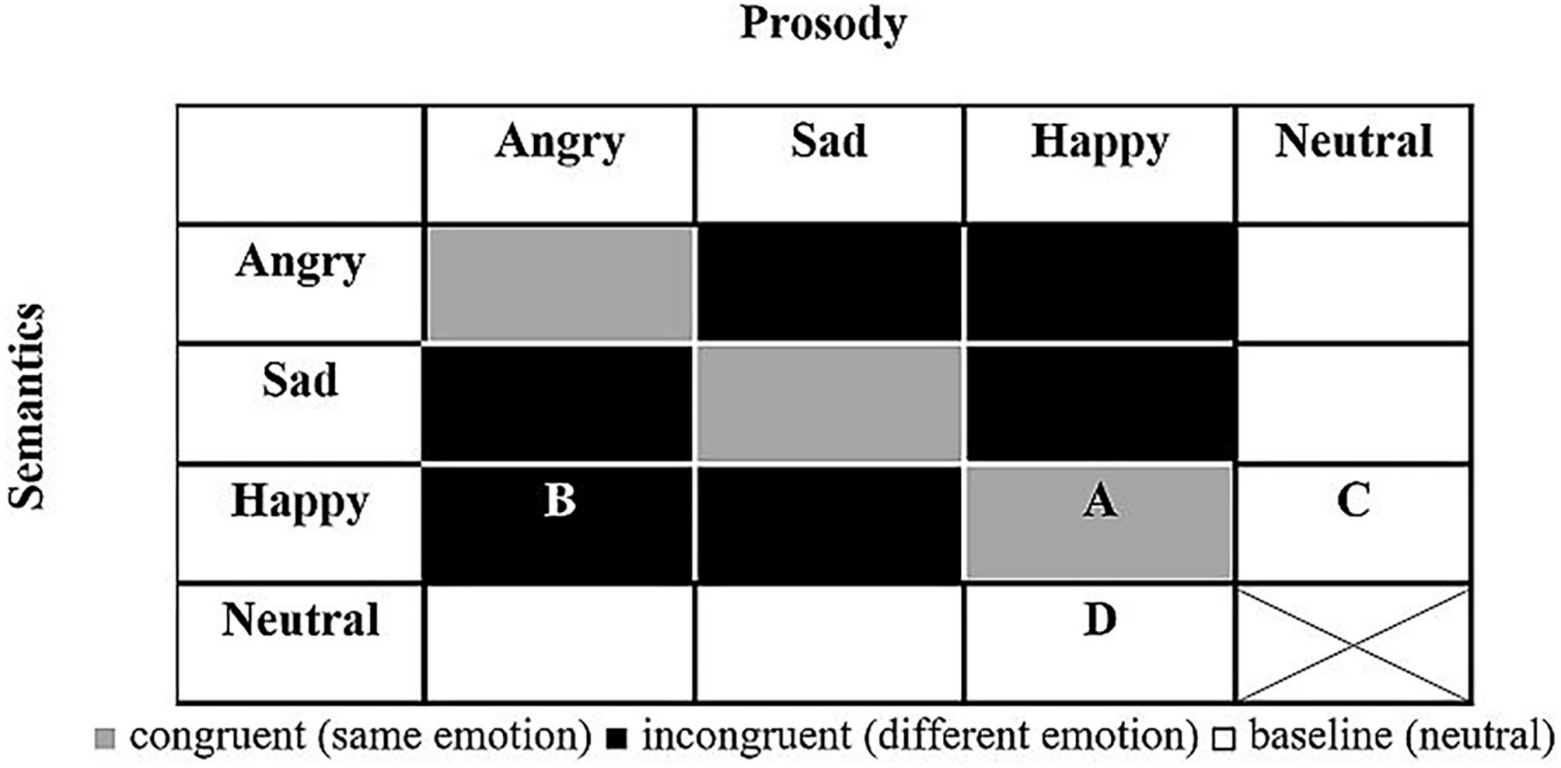
General design of T-RES stimuli. All combinations of prosody and semantics (16) are presented in each emotional rating block (note: neutral semantics spoken with neutral prosody was deemed uninformative and confusing and was not presented). A, example of congruent stimulus (happy semantics and happy prosody); B, example of incongruent stimulus (happy semantics and angry prosody); C, example of baseline semantics (happy semantics and neutral prosody); D, example of baseline prosody (neutral semantics and happy prosody).

**Figure 2:**
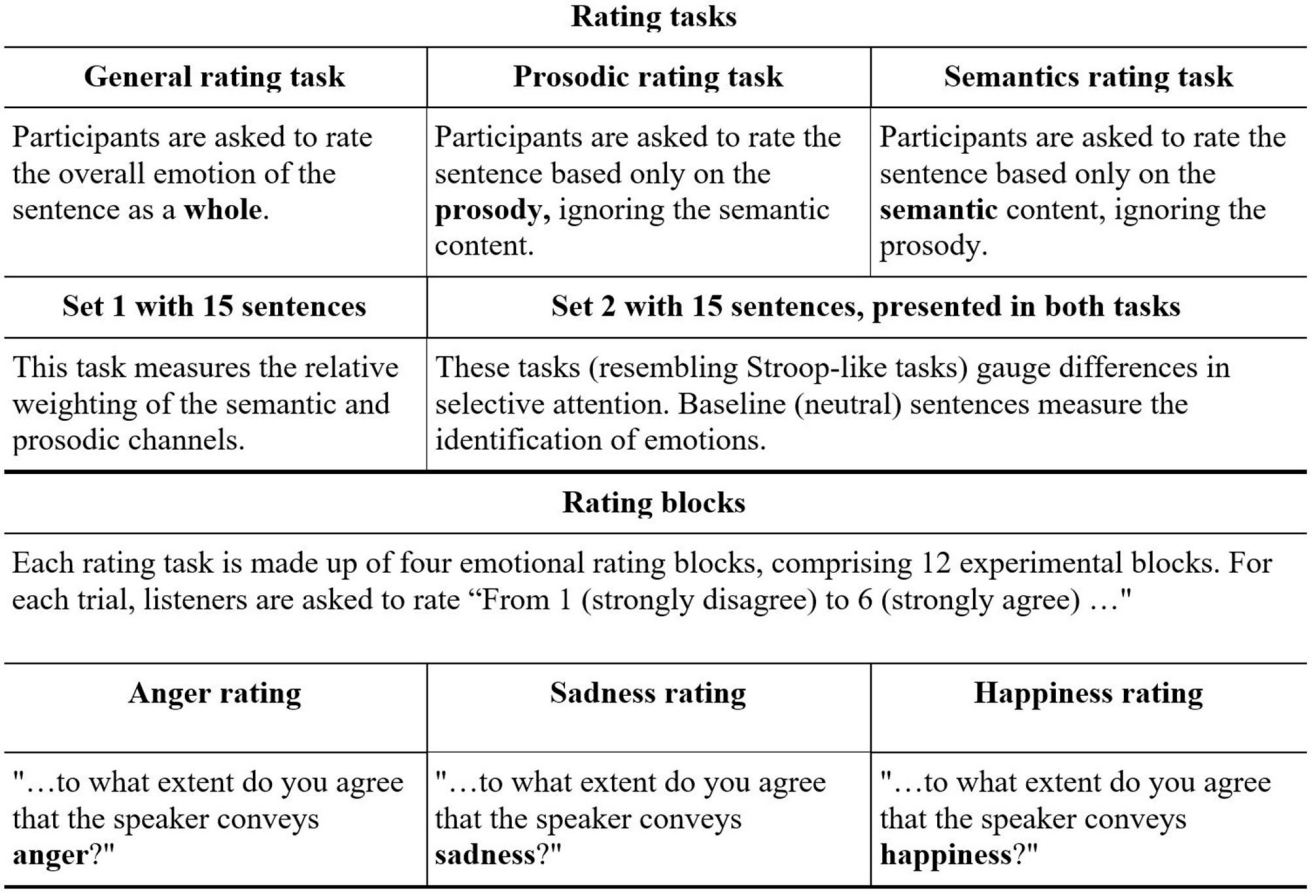
General design of T-RES: Rating tasks and rating blocks.

The current study aimed to test, for the first time, the perception of emotions in spoken language in people diagnosed with schizophrenia who committed severe violent offenses. To this end, the *Test of Rating of Emotions in Speech* [*T-RES*, (41)] was used to separately gauge the apprehension of semantics and prosody, and their relative roles in processing of spoken emotions, as depicted in Figures 1, 2.

In this test, participants listen to sentences that present emotional semantic and prosodic content in different combinations, both congruent and incongruent. In three separate tasks, listeners are asked to rate the extent to which they agree that a sentence conveys a predefined emotion, while focusing on either the semantic or the prosodic channel, or on both. The performance on each of these tasks directly tests three distinct components of processing of emotional speech: (a) *Identification* of emotions in the tone of speech (prosody) and semantics, (b) *Selective attention* by focusing on one channel while ignoring the other, and (c) *Integration* of the prosody and semantic content, thereby processing the spoken emotion sentence as a whole. The literature reviewed thus far led to the following predictions:

#### Impairment in Identification of Semantics and Prosodic Emotional Cues

Based on the literature, we hypothesized that forensic patients with schizophrenia would show impairments in identification of emotions presented in the semantic and prosodic channels—that is, assigning lower emotional ratings (i.e., less intense emotions) than their peers. To test this, performance on the baseline condition (in which one channel conveys neutrality) was gauged. For example, we tested whether forensic patients with schizophrenia would correctly identify the happy emotional *semantic content* of the sentence “I won the lottery today” spoken with neutral prosody. Similarly, we tested whether they would correctly identify the happy emotional *prosody* of the neutral semantic sentence “Red pipes are metallic” spoken with happy prosody (see white cells C and D in Figure 1). A group difference in these measures, if found, would suggest that forensic patients with schizophrenia process emotions in the prosodic or semantic channels differently than controls.

#### Failure in Selective Attention

We hypothesized that forensic patients with schizophrenia would fail to selectively attend to a specific channel (prosody or semantics) while actively ignoring the other, to a larger extent than controls. To test this, listeners were asked to rate the emotions presented in one channel (e.g., semantics) while ignoring the other channel (prosody) that conveys a different emotion (for semantic and prosodic rating of incongruent spoken sentences, see black cells in Figure 1).

#### Integration of Channels

In light of missing evidence in the literature, a hypothesis was not made as to whether forensic patients with schizophrenia would be less biased to the prosodic channel than controls when asked to integrate both prosodic and semantic channels. This was tested directly by the prosodic dominance measure, in which ratings of sentences that convey a designated emotion only in prosody are compared with those that convey this emotion only in semantics (for incongruent sentences, see black cells in Figure 1).

## Materials and Methods

The study received ethics approval from the medical center and two academic institutes affiliated with the authors. The study was carried out in accordance with the Declaration of Helsinki, and informed consent was obtained from all individual participants.

### Participants

The clinical group consisted of 21 male participants diagnosed with schizophrenia with a violent criminal record, who volunteered to participate with no monetary compensation (two additional participants had been excluded: one due to his age, 64 years, which exceeded the inclusion criteria; another failed to follow task instructions). They were recruited from the Maximum Secure Unit (MSU), a unique setting in a national mental health center in central Israel. All were under court-ordered compulsory hospitalization due to severe violent behaviors (including murder and rape). Based on the MSU's medical records (obtained by the MSU department heads), all had been diagnosed with ICD-10 schizophrenia (mean of duration from initial diagnosis = 8.6 years, *SD* = 6.6 years, range = 1–21 years), and nine of the 21 participants had reported a history of substance addiction prior to incarceration. All participants were stable, had no change to their treatment regimen during the last 4 months, and possessed the capacity to provide informed consent.

The control group consisted of 21 male volunteers from the general population that matched the clinical participants in socio-demographic characteristics (see Table 1). They were recruited by advertisements in and around the campus (including a local mall) and received the equivalent of $25 to compensate for their participation time.

**Table 1:** Participants' background data.

	**Clinical**	**Control**	***t*, *χ^2^***
N	21	21	
Age: mean (*SD*), years	36.3 (9.3)	34.3 (9.3)	*t*_(41)_ = 0.70, *p* = 0.49
Age: range, years	21-51	20-52	
Native Hebrew speaker	52%	57%	*$\chi_{\left(1\right)}^{2}$* =0.1, *p* = 0.76
Years of education: mean (*SD*)	11.5 (2.4)	12.1 (0.7)	*t*_(41)_ = 1.2, *p* = 0.23
Digit span: mean (*SD*)	4.8 (1.1)	6.1 (0.9)	*t*_(41)_ = 3.9, *p* < 0.001

#### Inclusion Criteria

Participants in both groups reported normal hearing (with no reported pathologies or history of hearing disorders), normal or corrected-to-normal vision, and no history of head trauma, neurological illness, or current substance use. To evaluate their basic cognitive auditory span, which may affect spoken language processing (77), the auditory forward digit span was administered to all participants, with the expected reduced performance for the clinical group (see Table 1).

### Measures and Tools: Test of Rating of Emotions in Speech (T-RES)

The Hebrew version of the T-RES (78) was used, with the following emotions: anger, happiness, sadness, and neutrality. The T-RES consists of three tasks. Two of the tasks relate to selective attention: (a) prosodic rating, in which listeners are requested to rate the emotion based only on prosodic information; and (b) semantic rating, in which listeners are requested to rate the emotion based only on semantic information. The third task was a general rating, an integration task in which listeners are requested to rate the emotion of the sentence as a whole. All spoken sentence stimuli had been pre-recorded by a professional female actress.

#### Stimuli

Figure 1 presents the makeup of the T-RES stimuli: the 15 spoken sentences in each semantic category are represented once in each of the tested prosodies, generating a 4 (semantic) × 4 (prosody) matrix. The cell marked “A” represents a congruent stimulus; e.g., a semantically happy sentence spoken with happy (congruent) prosody. Incongruent stimuli are represented by the cell marked “B”; e.g., a semantically happy sentence spoken with angry (incongruent) prosody. Baseline sentences present neutral content in one channel and emotional content in the other. In semantic baseline sentences, cell “C,” semantically emotional sentences (e.g., happy) are spoken with neutral prosody. In prosodic baseline sentences, cell “D,” semantically neutral sentences are spoken with emotional prosody (e.g., happy). For a full description of the characteristics of the spoken sentences and how they were constructed, see the research of Ben-David et al. (32, 42, 79)

#### Apparatus

The spoken sentences were presented on a 2.20 GHz Intel personal computer, using a 15.4-in. LCD monitor, via professional AKG K240 headphones, at a comfortable listening level (as confirmed by each participant). A research assistant was present throughout the experimental session, which lasted about 30 min.

### Procedure

Upon arrival, all participants received an explanation of the experimental tasks and those wishing to participate signed an informed consent form. The T-RES session was conducted only after participants were found to meet the inclusion/exclusion criteria. Subsequently, all participants were tested individually in a quiet room: the participants with forensic schizophrenia were tested in the MCU and the control participants were tested at the academic institute.

In the T-RES, each sentence is rated on three separate rating blocks, as depicted in Figure 2. For each trial, using a 6-point Likert scale, listeners are asked to rate “How much do you agree that the speaker conveys______ (anger, sadness, or happiness)? From 1—strongly disagree to 6—strongly agree.”

The experimental session began with the general rating task for all participants. For a randomly chosen half of the participants in each group, this was followed by the semantic rating task and then the prosodic rating task. This order was reversed for the other half of the participants. The order of the three emotion-rating blocks was counterbalanced by using the Latin square design, and the order of the trials in each block was fully randomized. In sum, each sentence was presented three times in each task, once in each of three rating blocks (anger, sadness, and happiness), with a total of 135 trials per session (conducted in under 25 min). The full description of the T-RES stimuli, design, and task is specified in previous works [e.g., (42)]. Reliability and validity of the tool are fully detailed in (32).

### Statistical Analyses

All of the following analyses used mixed-model repeated-measures ANOVAs (GLM) with average ratings as the dependent variable, Group (x2: forensic patients with schizophrenia vs. control) and Native Language (x2: native Hebrew speaker or not) as between-participants variables, and Target Emotion (x3: anger, sadness, or happiness) as a within-participants variable. Each test included one other within-participants variable. In prosodic- and semantic-rating tasks, Target Channel (x2: prosodic vs. semantic) was also used as a between-participants variable. Partial eta squared (η_*p*_^2^) was used as the measure for power in all statistically significant tests. As separate analyses did not find that criminally-related background characteristics (e.g., murder conviction and incarceration in a secure ward) impacted performance in the T-RES among the forensic patients with schizophrenia, they will not be further discussed.

## Results

### Identifications of Emotions Presented in the Prosodic and Semantic Channels

**Figure 3:**
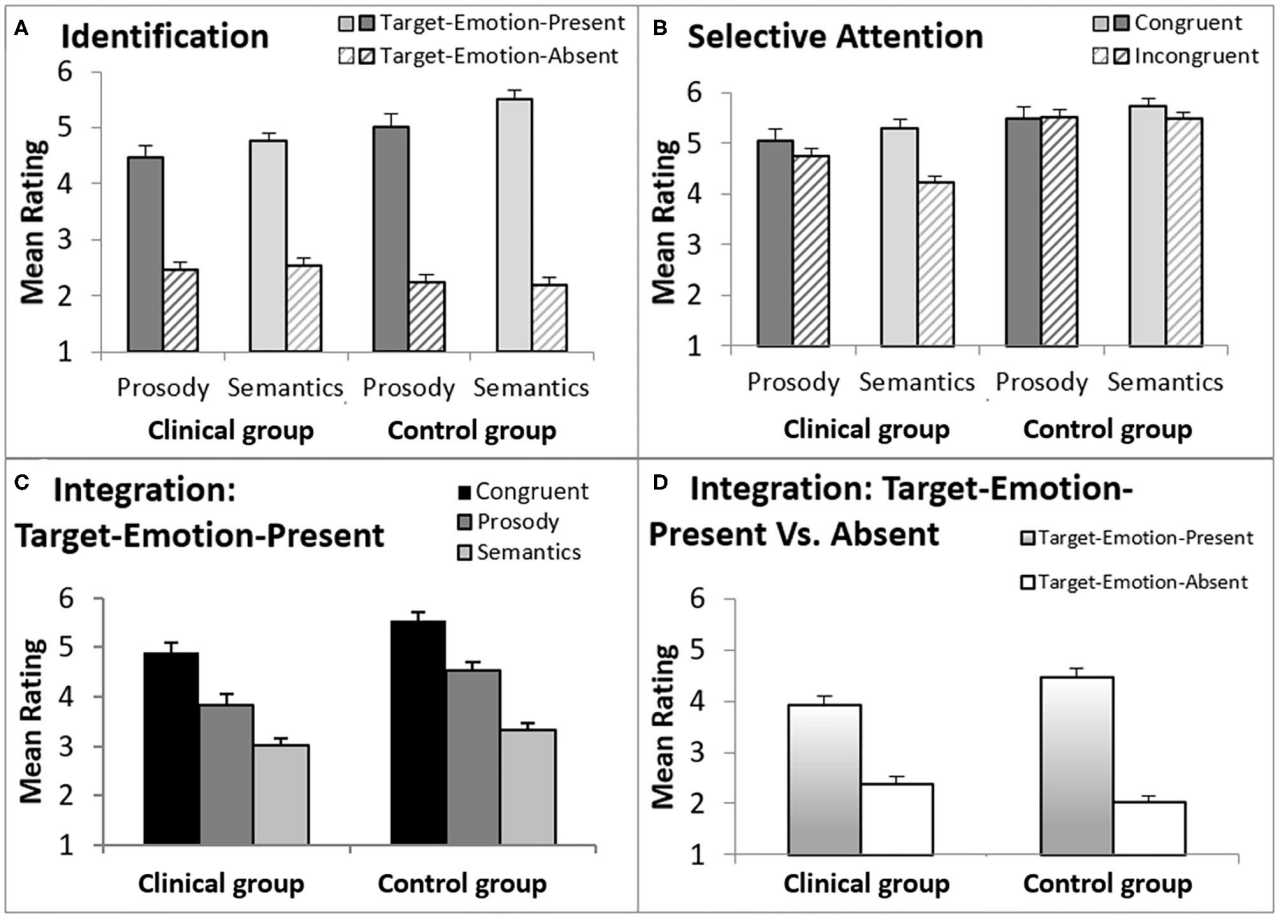
A graphic description of ratings in the T-RES tasks, separately for forensic patients with schizophrenia and controls. The error bars are standard errors of their respective means. **(A)** Identification, comparing target emotion-present and target-emotion-absent trials in the prosodic and semantic rating tasks; **(B)** Selective Attention, comparing congruent and incongruent trails, in the prosodic and semantic rating tasks; **(C)** Integration, presenting three types of target-emotion-present trials in the general rating task; **(D)** Integration, comparing an average of target-emotion-present trials with target-emotion-absent trials in the general rating task.

The first analysis tested whether both groups could correctly identify emotions in the prosody and semantic channels, respectively (prosodic- and semantic-rating tasks). This was tested in baseline sentences, when the to-be-ignored channel was neutral (represented by white cells in Figure 1). The tested variable was Emotion Identification, which was the difference between ratings of target-emotion-present trials (in which the target emotion was present in the attended channel) and target-emotion-absent trials (in which the target emotion was absent from the attended channel). The data is presented in the upper section of Table 2, and graphically displayed in Figure 3A.

**Table 2:** Summary of ratings (Means and *SD*s), averaged across target emotions, for the forensic patients with schizophrenia and the control group, with *F* values of the comparison.

	**Clinical**		**Control**		**Group effects**
**Identification (baseline sentences)**					
	**Prosody**	**Semantic**	**Prosody**	**Semantic**	
Target-emotion-present	4.5 (1)	4.8 (0.7)	5.0 (1)	5.5 (0.7)	
Target-emotion-absent	2.5 (0.7)	2.5 (0.6)	2.2 (0.7)	2.2 (0.6)	
Group X Identification (target-emotion-present vs. target-emotion-absent)					*F*_(1, 38)_ = 12.5, *p* = 0.001, *η_*p*_*^2^ = 0.25
**Selective attention**					
Congruent	5.1 (0.8)	5.3 (0.5)	5.5 (0.8)	5.7 (0.5)	
Incongruent	4.7 (0.7)	4.2 (0.7)	5.5 (0.7)	5.5 (0.8)	
Group X Selective Attention (congruent vs. incongruent)					*F*_(1, 38)_ = 14.5, *p* < 0.001, *$\eta_{p}^{2}$* = 0.28
**Integration**					
Congruent sentences	4.9 (0.8)		5.5 (0.8)		
Prosodic sentences	3.8 (0.6)		4.5 (0.6)		
Semantic sentences	3.0 (0.9)		3.3 (0.9)		
Group X Linear trend (congruent > prosodic > semantic)					*F*_(1, 38)_ = 1.0, *p* =0.32
Target-emotion-present *Average*	3.9 (0.5)		4.5 (0.5)		*F*_(1, 38)_ = 10.7, *p* = 0.002, *$\eta_{p}^{2}$* = 0.22
Target-emotion-present *Anger*	3.9 (0.7)		4.9 (0.7)		*F*_(1, 38)_ = 20.9, *p* < 0.001, *$\eta_{p}^{2}$* = 0.36
Target-emotion-present *Sadness*	4.0 (0.7)		4.7 (0.8)		*F*_(1, 38)_ = 7.7, *p* = 0.009, *$\eta_{p}^{2}$* = 0.17
Target-emotion-present *Happiness*	3.8 (0.5)		3.8 (0.6)		*F*_(1, 38)_ = 0.26, *p* = 0.61
Target-emotion-absent	2.4 (0.6)		2.0 (0.6)		*F*_(1, 38)_ = 3.61 *p* = 0.065, *$\eta_{p}^{2}$* = 0.09

A main effect for Emotion Identification was found, *F*_(1,38)_ = 379.7, *p* < 0.001, η_*p*_^2^ = 0.91, that significantly interacted with Group, *F*_(1,19)_ = 12.5, *p* = 0.001, η_*p*_^2^ = 0.25, indicating a larger effect for the control group than the clinical group (clinical group: *F*_(1,19)_ = 76.9, *p* < 0.001, η_*p*_^2^ = 0.80; control group: *F*_(1,19)_ = 794.7, *p* < 0.001, η_*p*_^2^ = 0.98). Target Channel (Prosody or Semantics), Target Emotion (Anger, Happiness, or Sadness), and Native Language (Native Hebrew speaker or Non-Native Hebrew Speaker) were each not found to generate a significant interaction with Group membership (clinical or control) and Emotion Identification (*F* < 1.3, *p* > 0.25).

In sum, the analyses indicated that both groups clearly identified the presented emotions in both prosody and semantics. However, participants in the control group were better able than the clinical group to distinguish between target-emotion-present (sentences that present the rated emotion in the target channel) and target-emotion-absent trials (sentences that do not present the rated emotion).

### Selective Attention to the Prosodic or the Semantic Channel

Selective attention was gauged by comparing average ratings of congruent sentences (presenting the rated-emotion *in both channels*) with incongruent sentences (presenting the rated-emotion *only in the target channel*), denoting the Selective Attention variable. The data is presented in midsection of Table 2 and graphically displayed in Figure 3B.

A significant main effect for Selective Attention, denoting failures of selective attention, was indicated, *F*_(1,38)_ = 29.3, *p* < 0.001, $\eta_{p}^{2}$ = 0.44, with larger failures found in the clinical group than in the control group (a significant interaction of Selective Attention and Group variables), *F*_(1,38)_ = 14.5, *p* = 0.001, $\eta_{p}^{2}$ = 0.28. A main effect for Group, *F*_(1,38)_ = 22.7, *p* < 0.001, $\eta_{p}^{2}$ = 0.37, indicated that the clinical group generally provided lower ratings (regardless of the stimulus type) than the control group. That is, averaged across congruent and incongruent sentences, forensic patients with schizophrenia gauged the rated emotion as less intense than controls.

Failures of selective attention were significantly higher when listeners were asked to ignore the prosody and focus on the semantics than vice versa (an interaction of Selective Attention and Target Channel) across both groups, *F*_(1,38)_ = 15.9, *p* < 0.001, $\eta_{p}^{2}$ = 0.30), and separately for the clinical group, *F*_(1,19)_ = 13.4, *p* = 0.002, η_*p*_^2^ = 0.41, but not for the control group, *F*_(1,19)_ = 3.0, *p* = 0.10 (see also a marginally significant triple interaction for Selective Attention, Group, and Target Channel, *F*_(1,38)_ = 3.84, *p* = 0.057, $\eta_{p}^{2}$ = 0.09). Target Emotion (Anger, Happiness, or Sadness) and Native Language (Native Hebrew speaker or Non-Native Hebrew Speaker) were each not found to generate a significant interaction with Group membership (clinical or control) and Emotion Identification (*F* < 0.35, *p* > 0.7).

To conclude, it appears that failures of selective attention were substantially more prominent for the clinical group than for the control group, with larger failures in inhibiting the prosodic than the semantic information.

### Integration of Channels and Channel Dominance

Figure 3C presents a graphic description of ratings of Trial Types in the general rating task, averaged across the three emotion rating blocks, separately for forensic patients with schizophrenia and control groups. From left-to-right, Figure 3C presents average ratings for congruent trials (the rated emotion appears in both channels), prosody trials (the rated emotion appears only in the prosody) and semantic trials (the rated emotion appears only in the semantics). There are two highly notable features of Figure 3C: (a) the similarity in the trend congruent > prosody > semantic trials in both groups; (b) higher ratings indicated by the control group, in all target-emotion-present trials (indicating more intense emotional ratings).

The statistical analyses supported these trends, with a significant linear trend (congruent > prosody > semantic) across groups, *F*_(1,38)_ = 164.8, *p* < 0.001, $\eta_{p}^{2}$ = 0.81, that did not interact significantly with Group membership, *F*_(1,38)_ = 1.0, *p* = 0.32. Across Trial Types and Target Emotions, the clinical group provided lower ratings than the control group, *F*_(1,38)_ = 10.7, *p* = 0.002, $\eta_{p}^{2}$ = 0.22. Notably, this effect of Group interacted significantly with the Target Emotion (Anger, Happiness, or Sadness), *F*_(2,76)_ = 11.1, *p* < 0.001, $\eta_{p}^{2}$ = 0.23. In other words, the clinical group provided lower ratings than the control group, indicating less intense emotional ratings, but the extent of this effect was dependent on the specific target emotion. In separate analyses conducted for each target emotion, the group difference in ratings was significant for the two negative emotions [Anger: *F*_(1,38)_ = 20.9, *p* < 0.001, $\eta_{p}^{2}$ = 0.36; Sadness: *F*_(1,38)_ = 7.7, *p* = 0.009, $\eta_{p}^{2}$ = 0.17), but not for the positive one (Happiness: *F*_(1,38)_ = 0.26, *p* = 0.61]. Additionally, Native Language did not interact with the linear trend or Group, nor did we find a significant interaction of the three (*F* < 1, *p* > 0.33 for all).

Finally, Figure 3D presents ratings for target-emotion-absent trials (the target emotion is absent from the semantics and the prosody) alongside target-emotion-present trials (average of target-emotion-congruent, prosody, and semantic trials). Analysis showed that discrimination, the difference between target-emotion-present and -absent trials, was reduced for the clinical group relative to the control group, *F*_(1,38)_ = 13.5, *p* = 0.001, $\eta_{p}^{2}$ = 0.26 (a significant interaction of Group and Trial Type).

In sum, the group of forensic patients with schizophrenia rated the negative emotions tested (Anger and Sadness) as less intense (lower ratings for target-emotion-present trials) than the control group. However, the positive emotion tested (Happiness) was rated as similarly intense (similar ratings for target-emotion-present trials) in both groups. In other words, the clinical group integrated the prosodic and semantic channels similarly to the control group, but under-rated the negative emotional information. Their ratings also indicated lower discrimination between target-emotion-present and target-emotion-absent trials—i.e., confusion in emotional ratings.

## Discussion

The present study aimed to examine the processing of emotions in spoken language (conveyed by the semantic and prosodic channels) in violent offenders diagnosed with schizophrenia. Three distinct components of auditory emotional processing were assessed: identification, selective attention, and integration. To this end, we used the T-RES, a tool dedicated to examining the processing of spoken emotions. The results indicated that forensic patients with schizophrenia successfully identified spoken emotions, but discriminated less effectively between emotions than controls. They also demonstrated larger failures to inhibit prosodic information while focusing on the semantics. Although they integrated the prosodic and semantic channels similarly to the controls, the forensic patients with schizophrenia under-rated negative emotional information (anger and sadness).

### Intact Identification of Emotions, but Reduced Discrimination

The findings of the current study indicate that forensic patients with schizophrenia were able to identify the presented emotions in both prosody and semantics. That is, ratings related to the degree of agreement that the target emotion was present were significantly higher (4.5–5.5) when indeed the target emotion was present (in either channel), than when it was absent (2.2–2.5). These results provide strong evidence to the preserved emotion identification abilities of forensic patients with schizophrenia, as the great majority of T-RES sentences (20 of 24) convey the target semantic emotions in an implicit manner (e.g., “You've won first place”), rather than explicitly, as tested in previous studies with this population [e.g., “I am happy to come dining with you” in (1)]. The current results were somewhat surprising, as deficits in identification of emotional prosodies [e.g., (9, 48)] and semantics (1, 53, 55) are considered well-known characteristics of schizophrenia.

Although identification of spoken emotions in the current study was intact for forensic patients with schizophrenia, they showed reduced ability to discriminate between emotions, relative to controls. Namely, their ratings indicated smaller differences between sentences that presented the rated emotions and sentences that did not (target-emotion-present vs. -absent). This pattern echoes previous findings (80) in which forensic patients with schizophrenia were better than non-forensic patients with schizophrenia at identification of facial emotional expressions, but less accurate at assessing their emotional intensity [for a similar effect with reduced feature discriminability in the presence of emotional words, see (81)].

### Larger Failures of Selective Attention and Prosodic Dominance

In the current study, forensic patients with schizophrenia were found to perform with substantially larger failures of selective attention than matched controls. As aforementioned, such failures have been previously documented in the auditory domain for patients with schizophrenia [e.g., (1, 55)]. The current study expands this evidence, for the first time, to the unique group of forensic patients with schizophrenia. Failure to inhibit irrelevant auditory information (e.g., an emotional cue available in a social interaction) may lead to deficits in behavioral regulation, impulse control, and aggressive behaviors (58, 72). This, in turn, may lead to the criminal behavior that has been documented in forensic patients with schizophrenia.

Methodologically, it is also noteworthy that the majority of previous studies that found selective attention deficits in forensic patients with schizophrenia used neuropsychological tasks (e.g., Stroop, Go-no-go). In contrast, the current study showed similar evidence using an ecological task that mimics daily social behavior—the processing of emotions in spoken sentences. Therefore, increased failures of selective attention, as documented in the current study, can be more easily generalized to daily life situations for forensic patients with schizophrenia.

Failures of selective attention found in the current study were more prominent when the clinical participants were asked to inhibit the prosodic than the semantic information. This may hint that the prosodic channel is more dominant than the semantic one, when the task calls for selective attention. A prosodic bias may indeed be related to violent behaviors. Consider, e.g., the semantically neutral everyday sentence “Hi neighbor, could you please place the garbage in the container?” spoken with a stern, serious prosody. As violent offenders may display a “hostile attribution bias,” a tendency to view neutral expressions and behaviors as hostile [(82); for a review, see (26)], failing to inhibit the (negative) prosodic cues may lead to inappropriate social reactions for forensic patients with schizophrenia [see also (83)]. Indeed, poor executive functioning (e.g., inhibition) has also been associated with the risk of aggressive-behavior recidivism in schizophrenic patients (72).

### Preserved Integration of Prosodic and Semantic Information, but Under-Rating of Negative Emotions

The current study is the first to demonstrate a preserved ability of forensic patients with schizophrenia to integrate emotional information presented in two separate auditory channels: prosody and semantics. As deficits in multisensory integration are common in schizophrenia (74–76), the current data may suggest that performance is preserved when uni-sensory (auditory) integration is called for. This preserved ability has clinical importance, given that the stimuli used by the T-RES are spoken sentences rather than single words [e.g., (53)]. This may be especially challenging, considering the attentional and verbal working memory deficits often reported in this population (84) and documented in the current study (see digit span data in Table 1). The presence of underlying challenges in these executive functions amplifies the strength of the finding of preserved (uni-sensory) channel integration.

One of the indicators of preserved integration is congruency supremacy (41). Indeed, in the current study, congruent sentences (which present the same emotion in both channels) received higher emotional ratings (indicating the most intense emotion) than all other rated-emotion-present trials (prosodic and semantic trials) among both groups, replicating previous findings with the T-RES paradigm. This effect somewhat echoes previous findings on schizophrenia by Brazo et al. (1). In their study, although individuals with schizophrenia were less accurate than their matched controls at categorizing spoken sentences conveying emotion, they benefitted from the redundancy of information in sentences with congruent prosody-semantics more than controls [for a discussion of redundancy gains in congruent presentation, see (85, 86)].

Interestingly, in the present study, forensic patients with schizophrenia under-rated negative (spoken) emotional information, somewhat in contrast with evidence in the literature on a negative bias in recognition of facial expressions [visual information; see (31)]. In our data, when asked to rate anger or sadness, the clinical group provided lower ratings than their peers, but no such differences were found for the positive emotion. For example, when asked to rate a spoken sentence that conveys happiness and anger in different channels, the clinical group provided lower anger ratings than the control group, while no significant group differences were documented for happiness ratings. This may suggest that forensic patients with schizophrenia have specific difficulties in processing spoken negative affect, unlike spoken positive affect. A study by Klumpp et al. (87) similarly found that, among patients with schizophrenia, negative semantics elicited a unique evoked response potential (N400) that did not occur with positive semantics.

Alternatively, one may relate the reduced ratings in emotional discrimination and integration that was documented for the clinical group as reflecting a flat effect—an experience of reduced emotional intensity, a well-known schizophrenia symptom [for a discussion, see (88)]. However, if forensic patients with schizophrenia were to show a flat affect, then lower ratings should have been reflected on *all* emotional rating scales. As the current data indicated lower ratings *only* on the negative emotions (see Figure 3), our findings do not appear to support the notion of a flat affect effect among the clinical group.

### Caveats and Future Directions

A possible limitation of the current study concerns the clinical sample that included only male offenders. However, males represent the majority of offenders in secure mental wards (89). There is also evidence in the literature to suggest that males may be especially susceptible to dysfunction in emotional processing, whereas recognition of affective prosody and emotional semantics may be preserved in females [e.g., (55)]. Future studies may wish to include female offenders as well, evaluating possible gender differences. In addition, as the subgroup of forensic patients with schizophrenia differs from non-violent patients with schizophrenia, further studies should compare performance between the two groups.

A few limitations also relate to the T-RES instrument itself. First, the sentences were recorded by one professional female actress, rather than different speakers. Although this may potentially decrease the generalizability of the data, we argue that this also minimizes confounding factors. Second, the current study tested only Hebrew speakers. Since the perception of emotions in speech may be affected by cultural variables (90, 91), future studies may wish to examine the validity of the results when testing individuals from various cultures (or languages) with appropriate stimuli (92). Third, the T-RES evaluates the processing of basic and concrete emotions. Possibly, group differences may be more pronounced if more abstract and complex emotions (e.g., boredom, envy) would be tested. Future studies may wish to examine the processing of such emotions as well.

### Clinical Implications

The current study's results may be useful to guide new rehabilitation approaches matched to the pattern of auditory emotional processing presented by forensic patients with schizophrenia. Forensic patients with schizophrenia may respond poorly to verbally-mediated treatment programs, as they processes spoken emotions differently than intended by the speaker. This should be acknowledged by the therapist. Moreover, targeted programs could focus on remediation of difficulties in discrimination between emotions, failures in inhibiting prosodic information, and the tendency to under-rate negative emotional information. These programs could use explicit or implicit methods to train participants to pay attention to emotional features they may have missed**;** relying on the preserved abilities of forensic patients with schizophrenia to identify spoken emotions and to integrate the semantic and prosodic speech channels. For example, we suggest tailoring Social Cognition Training Programs, which have been found to show promise in improving prosodic-affect recognition in schizophrenia [for reviews, see (93, 94)].

The results also support the use of the T-RES as a sensitive tool in identifying the nuances of components underlying the processing of spoken emotions in various clinical populations. Recently, in response to COVID-19 social restrictions, a remote adaptation (an online version) of the T-RES has been validated, iT-RES (95), increasing the feasibility of the test. We suggest incorporating the iT-RES to the arsenal of assessment tools for forensic patients with schizophrenia, to better portray idiosyncratic emotion processing performance, even in tele-health. As suggested by Leshem et al. (26), identifying difficulties in spoken emotion processing might also assist in prevention of recidivism in forensic populations.

## Data Availability Statement

The raw data supporting the conclusions of this article will be made available by the authors, without undue reservation.

## Ethics Statement

The studies involving human participants were reviewed and approved by IRB, Shaar Menashe Mental Health Center IRB, Psychology, Bar-Ilan University IRB, Psychology, Interdisciplinary Center (IDC) Herzliya. The patients/participants provided their written informed consent to participate in this study.

## Author Contributions

The manuscript was written by RL, MI, and BB-D. Research design was conducted by RL and BB-D. Data was collected under the supervision of RB, RL, and BB-D. Data analysis was conducted by BB-D. BB-D was the corresponding author for the paper. All authors contributed to the article and approved the submitted version.

## Conflict of Interest

The authors declare that the research was conducted in the absence of any commercial or financial relationships that could be construed as a potential conflict of interest.
